# HOW CAREGIVERS AND OLDER US VETERANS MANAGED SOCIAL ISOLATION DURING THE COVID-19 PANDEMIC

**DOI:** 10.1093/geroni/igac059.1963

**Published:** 2022-12-20

**Authors:** Leah Haverhals, Roman Ayele, Hillary Lum

**Affiliations:** VA Eastern Colorado Health Care System, Rocky Mountain Regional VA Medical Center, Denver, Colorado, United States; VA Eastern Colorado Health Care System, Aurora, Colorado, United States; University of Colorado Anschutz Medical Campus, Aurora, Colorado, United States

## Abstract

To protect themselves from contracting the SARS-CoV-2 virus, many older adults managing multiple medical conditions experienced increased social isolation. The objective of our qualitative research study was to describe how older Veterans receiving care from the United States (US) Department of Veterans Affairs (VA) healthcare system, and their caregivers, managed increased social isolation during the pandemic. We recruited Veterans and their caregivers residing in rural and urban areas who received care from either a tele-palliative care or a tele-geriatrics clinic connected to one VA Medical Center, inviting them to participate in phone interviews. From May-September 2021, we interviewed N=23 participants (n=9 Veterans and n=14) caregivers. We applied a deductive and inductive approach to thematic analysis to analyze interview data. Findings revealed that while caregivers experienced increased anxiety, which they attributed to pandemic-related changes, they also expressed solidarity in that others were experiencing similar stressors. Many caregivers and Veterans shared experiences of increased loneliness, which some found difficult to manage as communication with their social networks was sparse. At the same time, the pandemic made them value relationships with others more than before. Some Veterans noted they kept busy with hobbies and did not feel much loneliness despite increased isolation. Caregivers caring for Veterans with dementia stated they experienced confusion about their narrower social networks because they could not remember reasons why they were not regularly spending time with them. Findings demonstrate the need to identify strategies and policies to better support caregivers and older Veterans during times of crisis.

